# Bioequivalence study between two formulations of ciclosporin A (Cyclavance® oral solution and Atopica® soft capsules) following a single oral administration to dogs

**DOI:** 10.1186/s12917-016-0669-9

**Published:** 2016-03-12

**Authors:** C. Navarro, L. Séguy, M. Vila, P. Birckel

**Affiliations:** Virbac, Medical Department, Carros, France; Revest les Roches, France; AmatsiAvogadro, Bioservices department, Fontenilles, France

**Keywords:** Dog, Ciclosporin, Cyclosporine A, Bioequivalence, Pharmacokinetics, Substitution, Interchangeability, HPLC

## Abstract

**Background:**

Ciclosporin is a selective immunomodulator used for the treatment of atopic dermatitis in dogs*.* A new 100 mg/ml oral solution formulation (Cyclavance®, Virbac) was developed as a pharmaceutical equivalent to the marketed capsule formulations (Atopica®, Novartis Animal Health) containing 25, 50 mg, or 100 mg of ciclosporin A. The aim of this study was to assess and compare the pharmacokinetic profiles and bioequivalence of the two formulations following a single oral administration to dogs. This randomised, two-period, two-sequence, crossover bioequivalence study was conducted in 40 healthy dogs under fasting conditions. Each dog received either one 50 mg capsule of Atopica® or 0.5 ml of Cyclavance®. After dosing, blood samples were collected during a 48-h time period at 0, 0.5, 1, 2, 4, 6, 12, 24, 36 and 48 h. Blood ciclosporin A concentrations were measured by using an HPLC-MS/MS method. Cmax, Tmax, t1/2, AUC0-t, AUC0-∞ and Kel were determined for the two ciclosporin formulations. Bioequivalence was to be concluded if the 90 % confidence intervals were within the range of 80 % to 125 % for Cmax and AUC0-t. Dogs were monitored once daily throughout the study period for adverse effects.

**Results:**

The 90 % confidence intervals for Cyclavance®/Atopica® mean ratios of the log-transformed pharmacokinetic variables Cmax and AUC0-t were within the conventional bioequivalence range of 80 % to 125 % (Point estimate: 101.2 % and 101.4 % respectively). Except for salivation reported after administration of both products, or vomiting and diarrhoea reported after Atopica® administration, both formulations were well tolerated in the 40 healthy dogs over the 48-h study period.

**Conclusions:**

The two ciclosporin oral formulations demonstrated similar pharmacokinetic profiles and were found to be bioequivalent, and therefore, interchangeable.

## Background

Ciclosporin is a calcineurin inhibitor with potent immunosuppressive and immunomodulatory activities resulting from decreased production of interleukin-2 and proliferation of T-cells [1, 2]. It reduces the number and activity of proinflammatory cells which induce activation of cells initiating the cutaneous immune response (Langherans’ cells and lymphocytes) and cells mediating allergic reactions (mast cells and eosinophils). As a result to this inhibition, ciclosporin exhibits strong anti-allergic effects and anti-inflammatory activity [3, 4].

Ciclosporin has been used in veterinary dermatology for the control of allergic chronic diseases for more than a decade [2]. A growing number of studies have investigated the efficacy and safety of ciclosporin in the treatment of canine atopic dermatitis [1–3, 5–8].

The International Task force on Canine Atopic Dermatitis 2010 practice guidelines concluded that there was good evidence for high efficacy of ciclosporin at 5 mg/kg once daily in the treatment of dogs with atopic dermatitis [6].

Atopica® soft capsules, a tablet formulation of ciclosporin A available in 25, 50, and 100 mg soft gelatin capsules, is approved for use in canine atopic dermatitis and has been available since 2002. Previous pharmacokinetic studies on Atopica® soft capsules in dogs, have shown that ciclosporin is rapidly absorbed, principally from the small intestine [1, 2], with mean peak blood concentrations achieved 1–2 h after oral administration [9]. Bioavailability is however variable and can range from 23 % to 45 % [1, 2]. This low and highly variable absolute oral bioavailability can be explained by the high molecular weight of the drug, its low water solubility, the effect of the P-glycoprotein efflux pump at the intestinal level, and metabolism by cytochrome P450 3A enzymes located in the small intestinal mucosa and liver [1, 2, 8]. Furthermore, because the bioavailability of ciclosporin in dogs decreases when given with food, the recommendations are to administer the drug 2 h before or after feeding [1, 2]. Being lipophilic, ciclosporin distributes widely in the tissues and its concentration in epidermis and dermis is about 10-fold higher than in the blood [1, 2, 8]. Elimination is mainly biliary with minimal renal excretion [1, 2, 8]. The relatively long half-life of the drug (8.6 h) [9] and its concentration in the skin after oral administration [10] support once daily dosing in dogs.

A new oral liquid formulation (Cyclavance®) of ciclosporin A is now available for dogs in Europe and facilitates precise dosing and owner compliance [11]. In human medicine, studies comparing the bioequivalence and pharmacokinetic conversion of Neoral® product (Novartis, original formulation) with that of Equoral® product (Teva, generic formulation) have demonstrated that these drugs are bioequivalent and interchangeable in stable patients [4, 8].

The objective of this study was to compare the pharmacokinetic profiles of Cyclavance® oral solution and Atopica® soft capsules dosed on an empty stomach in order to evaluate their bioequivalence and consequently the possibility of substitution between the two drugs in dogs.

## Methods

### Animals

The study was performed on 40 male, adult and healthy beagle dogs. The animal bodyweight ranged from 8.775 to 10.860 kg. The dogs were housed in pens and fed daily with ration of an adapted pelleted feed, with access to water *ad libitum*.

Housing room temperatures were recorded daily and ranged from 13.8 °C to 21.4 °C.

All animals were managed similarly with due regard for their well-being according to prevailing practices (Directive 86/609/EEC).

### Formulations

The commercially available reference product containing ciclosporin A, Atopica® 50 mg soft capsules for dogs (Novartis Animal Health.) and Cyclavance® oral solution containing the same active ingredient, ciclosporin A 100 mg/ml oral solution (Virbac SA) were used to carry out the current study.

### Study design

The study was conducted in a single dose, randomised, two-period, two-sequence, cross-over design with a 8-day washout period between treatment.

The 40 animals were randomly allocated into two treatment groups: a single dose of 50 mg ciclosporin capsules (Atopica®) or 0.5 ml oral solution (Cyclavance®) was administered to the fasted dogs.

After dosing, blood samples were collected during a 48-h period. Blood samples of 2 ml each were drawn at 0, 0.5, 1, 1.5, 2, 4, 6, 12, 24, 36 and 48 h after administration.

Animals were fasted at least 15 h before each treatment and were fed after the 4-h post-treatment sampling. Animals had free access to water. Animals were observed once daily for any adverse events.

The project was evaluated and approved by the ethical committee of AmatsiAvogadro. It was conducted in accordance with the principles of Good Laboratory Practices (EC principles of Good Laboratory Practices, Directives 2004/10/EC of the European Parliement) and according to Guidelines for the conduct of pharmacokinetic studies in target animal species (EMEA/CVMP/133/00-FINAL) and Guidelines for the conduct of bioequivalence studies for veterinary medicinal products (EMA/CVMP/016/00-Rev.2).

### Analytical method

Blood ciclosporin A concentrations were determined using an HPLC-MS/MS method previously validated for specificity, sensitivity, linearity, recovery, precision, accuracy, stability. A mixture of red blood cells and proteins in 0.1 mL blood fortified with internal standard by addition of 0.2 mL 0.1 % formic acid in acetronitrile was vortexed. The mixture was vortexed again after addition of 0.4 ml of ammonium acetate (20 mM) and 3 ml of tert-butyl-methyl-ether. The supernatant was evaporated to dryness and the residue dissolved in 0.2 ml of reconstitution solvent. Then the sample was assayed by reversed phase HPLC with methanol/water (gradient over 12 min) as mobile phase, using a C18 column, detection by MS/MS (MRM transition: 602.2 > 100.1) and quantitation by peak area using ciclosporin A -D12 as internal standard. The calibration curve was prepared with fortified blood specimens of untreated animals combined with the internal standard and enabled the recovery-corrected determination of ciclosporin A in unknown specimens. The calibration curve performed from the assay ranged from 10 ng/ml (Lower limit of quantification: LLOQ) to 2000 ng/ml (Upper limit of quantification: ULOQ). The validation of the method yielded a mean recovery of 76.6 % and a precision with a coefficient of variation of 5.0–8.0 % for repeatability. Matrix effect was not observed. Only concentrations equal or exceeding the LLOQ were used.

### Pharmacokinetic and statistical analysis

The sample size was calculated on the basis of a cross-over design with log-transformed data according to Hauschke et al. [12], considering an intra-individual variation coefficient of 22.7 %, a power of the test of 80 %, a confidence interval of 80–125 %, an expected ratio μT/μR (mean of test versus reference) of 1.10, based on Steffan et al. [13].

Under these conditions, the calculated sample size was 40 animals.

The highest blood concentration observed and the corresponding time was defined as the Cmax and Tmax values, respectively. The terminal rate constant (Kel) was obtained by linear regression of the log-linear terminal phase of concentration-time profile using at least 3 consecutive data points and starting from the last non zero value. The terminal half-life (t1/2) was obtained as ln2/Kel. AUC0-t (area under the curve between time 0 and time of the last quantifiable value) was calculated by the mixed log-linear method. The extrapolated AUC (AUC0-∞) was calculated from the computed last quantifiable blood concentration divided by Kel and the percentage of extrapolated AUC (%AUC0-∞) was obtained using the following formula: %AUC0-∞ = (AUC0-∞/(AUC0-∞ + AUC0-t)) x100.

The pharmacokinetic parameters were generated using WinNonlin software version 6.1 (Pharsight).

In accordance with the general recommendations, the analysis of variance was performed after logarithmic transformations of the parameters AUC0-t and Cmax. A statistical comparison of the pharmacokinetic parameters Cmax and AUC0-t was performed by a mixed effects model analysis. Alpha risk was of 0.5 %.

For the pivotal parameters AUC0-t and Cmax, bioequivalence was determined when the 90 % confidence interval of these two parameters were within 0.8 and 1.25.

## Results

Salivation was observed for 6 animals (5 receiving Cyclavance® oral solution and 1 receiving Atopica® soft capsules) on period 1. For one animal, the effect was observed before and after administration of the product. Similar observations were done on three animals (2 receiving Cyclavance® oral solution and 1 receiving Atopica® soft capsules) on period 2. As salivation was observed before treatment and was less frequent in period 2, it was concluded that it was due to the stress of animals at the start of the study. One animal vomited 29 min after administration of Atopica® soft capsules. The empty capsule was found in the vomit. The animal was not excluded from the study. Diarrhea was observed in the box of one animal receiving Atopica® soft capsules at 30 min sampling time. No other abnormal findings were observed on animals during the study. Bodyweights of the dogs did not differ significantly over the study duration (9.754 kg and 9.697 kg for the dogs receiving Atopica® soft capsules and 9.668 kg and 9.568 kg for the dogs receiving Cyclavance® oral solution, respectively on D0 and D8).

**Fig. 1:**
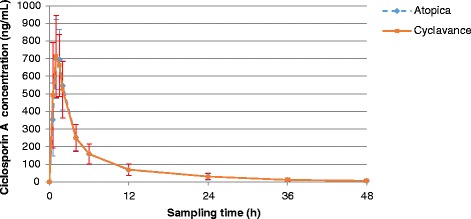
Mean ciclosporin A concentrations obtained in dog blood (with linear scale)

**Fig. 2:**
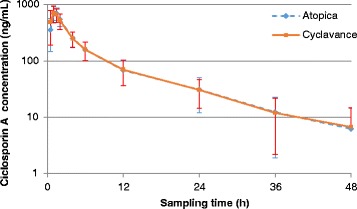
Mean ciclosporin A concentrations obtained in dog blood (with semi-log scale)

Blood concentrations are presented in Table 1 and illustrated in Figs. 1 and 2. For the mean concentration calculation, BLQ values were set as 0. The pharmacokinetic parameters obtained after a single oral administration of Atopica® soft capsules or Cyclavance® oral solution are presented in Table 2. The pharmacokinetic parameters were not dose normalized. Consequently, AUC and Cmax were not divided by the administered dose. The low extrapolation for AUC0-∞ demonstrated that sampling times were adapted, and therefore, that the terminal half-life was relevant of the elimination of ciclosporin A.

**Table 1:** Mean ± SD ciclosporin A concentrations (ng/mL) obtained in dog blood

Theoretical sampling time (h)	Atopica® soft capsules		Cyclavance® oral solution	
0	BL/Q		BLQ	
0.5	354.18	±206.45	491.54	±300.01
1	697.09	±224.49	712.68	±231.95
1.5	695.48	±169.80	662.00	±176.35
2	547.09	±139.04	524.47	±160.45
4	251.56	±73.61	250.15	±76.41
6	158.27	±56.38	158.55	±56.26
12	68.24	±31.77	69.86	±33.72
24	30.97	±18.92	30.65	±16.29
36	12.29	±10.40	11.96	±9.75
48	6.21	±8.24	6.73	±7.92

*BLQ* below the lower limit of quantification

**Table 2:** Descriptive statistics for pharmacokinetic parameters

Treatment type		k_el_	t_1/2_	T_max_	C_max_	AUC0-t	AUC_tot_	% AUC _0-∞_
		(1/h)	(h)	(h)	(ng/mL)	(h.ng/ml)	(h.ng/ml)	(%)
Atopica® soft capsules	Mean	0.076	NA	1.24	772.18	3680	3901	5.8
	SD	0.037	NA	0.32	163.8	1263	1336	1.4
	CV%	49.4	NA	25.6	21.2	34.3	34.3	23.3
	Harmonic Mean	0.066	9.13	1.16	734.53	3279	3489	5.5
	Min	0.044	3.23	0.50	361.39	1236	1334	3.5
	Max	0.214	15.75	2.00	1121.98	7763	8393	8.5
Cyclavance® oral solution	Mean	0.073	NA	1.12	786.97	3728	3955	5.8
	SD	0.026	NA	0.36	188.3	1243	1305	1.3
	CV%	35.7	NA	32.5	23.9	33.3	33	22.3
	Harmonic Mean	0.064	9.55	1	736.31	3339	3549	5.6
	Min	0.030	5.56	0.50	334.61	1654	1740	3.7
	Max	0.125	23.08	2.00	1106.28	6907	7170	9.8

The upper and lower limits of the calculated confidence intervals are presented in Table 3.

**Table 3:** Upper and lower limits of the calculated 90 % CI

Pharmacokinetic parameters	90 % confidence interval			CV_W_ (%)
	Lower limit	Upper limit	Point estimate	
**C**_max_	96.2894 %	106.2942 %	101.1682 %	13
AUC_0-t_	96.2139 %	106.9190 %	101.4253 %	14

Analysis of bioequivalence between both products showed that 90 % Confidence Interval for Cmax and AUC0-t were within 80 % and 125 % (96.29 % and 106.29 % for the Cmax and 96.21 % and 106.92 % for the AUC0-t). No sequence, treatment and period effects were observed for each studied pharmacokinetic parameters.

## Discussion

As defined by the European Medicines Agency [14], bioequivalence techniques are scientific methods for the comparison of different veterinary medicinal products containing the same active substance. Bioequivalence testing aims to demonstrate that two medicinal products produce plasma concentrations similar enough to conclude that the systemic effects of the two products, in respect to efficacy (and possibly safety), are the same. According to the guidance specifying requirements for the design, conduct, and evaluation of bioequivalence studies for veterinary medicinal products with systemic action [14], generics should be tested against Brand in healthy animals of the target species and from a homogeneous group, by giving a single dose of the reference formulation and the generic formulation that is being tested. Bioequivalence studies should be performed using AUC and Cmax to demonstrate that the generic has similar pharmacokinetics as the brand formulation. AUC serves as a surrogate for the extent of absorption whereas the Cmax and the time of its occurrence (Tmax) together characterize the rate of absorption. Two veterinary drugs are considered bioequivalent if the upper and lower limits of the 90 % confidence interval of the generic-to-brand ratio for AUC0-t and Cmax falls within the range 80–125 % [14].

Conducted in healthy dogs and in a single dose, randomised, two-period, two-sequence, cross-over design with a 8-day washout period between treatment, the present study met applicable standards for bioequivalence studies in animals [14]. Furthermore, sampling times chosen in accordance with the EMA guidelines and the use of a previously validated LC-MS/MS method for measuring blood ciclosporin A levels, enabled to accurately characterize the plasma concentration-time profile of the two drugs. Indeed, LC-MS/MS has been described as one of the most sensitive, reliable and fast analytical technique for bioequivalence studies [15, 16] and is commonly used for determining ciclosporin A levels [1]. When Cyclavance® oral solution and Atopica® soft capsules were administered orally at a target dose of 5 mg/kg body weight of ciclosporin A, the parametric 90 % confidence intervals of the mean ratio test/reference were actually included within the reference confidence interval [0.80–1.25] for Cmax and AUC0-t parameters. Otherwise, the values of the pharmacokinetics parameters observed in this study for Atopica® soft capsules are close to those reported in previous studies using an HPLC assay [9, 13]. The mean Tmax value was 1.24 h compared to 1 to 2 h mentioned by Guaguère et al. [9] and the mean terminal T_1/2_ value of 9.13 h found in the present study was within the range (5–11 h) described by Guaguère et al. [9]. The Cmax value was 772.18 ng/mL slightly above the value reported by Steffan et al. [13] (577 ng/mL). The statistical analysis was performed with all animals, including animal for which vomit was observed 30 min after the administration of Atopica® soft capsules. For this animal, the pharmacokinetic profile of Atopica® soft capsules seemed to be altered by this observation since Cmax and AUC values were lower after administration of Atopica® soft capsules than after administration of Cyclavance® oral solution. However, the inclusion of this animal in the statistical analysis did not affect the assessment of the bioequivalence between both products.

Although 40 dogs were used in a cross-over design in this study, blood concentrations of ciclosporin A showed quite large inter-animal variations during the 48-h period following drug administration. Indeed, standard deviations varied between 8.24 and 224.49 in the dogs receiving Atopica® soft capsules and between 7.92 and 300.01 in the dogs receiving Cyclavance® oral solution. Such variations had already been found in a clinical trial on perianal fistulas as well as in another pharmacokinetic study [13]. Many other studies however indicated that drug absorption and metabolism were relatively constant in healthy dogs [9, 13]. Even if the reasons for this discrepancy could not be identified [13], they may be explained in part by the relatively low bioavailability of ciclosporin A due to the large molecular mass of the drug, its low water solubility, as well as its partially metabolization and limited absorption in the intestines [9]. In dogs, the bioavailability of the drug administered orally as a vegetable oil-based formulation is in the range 20–27 % whereas dedicated formulations to dogs offer a 35 % bioavailability [9]. As a consequence, by increasing absolute bioavailability and reducing biliary secretion, specific dog ciclosporin A formulations tend to decrease the interindividual variability of drug absorption [9].

In man, ciclosporin has a <2-fold difference between the minimum toxic concentration and minimum effective concentration in blood [17, 18], and the debate as to whether or not it is necessary to apply stricter guidelines for such narrow therapeutic index drugs has been ongoing for several decades in human health. Indeed, it has been suggested that the usual acceptance interval for AUC and Cmax may need to be tightened to 90 % to 112 % for such drugs, but there is currently no international consensus on the subject [17, 18]. However, even if the pharmacokinetic properties of ciclosporin are very similar in dogs and man, its safety margin is much wider in dogs [9]. Therefore, more restrictive criteria are not necessary for assessing bioequivalence of ciclosporin formulations in dogs. Nevertheless, the results of the present study show that the criteria proposed in man were largely met anyway since the intervals were 96.29 to 106.29 % for Cmax and 96.21 to 106.92 % for AUC0-t (Table 3).

In human health, the scientific aspects of bioequivalence that govern the use of generics are sometimes described ambiguously in the literature, and they are therefore not always perceived clearly by health professionals. This lack of clarity may be an obstacle to their use [17]. However, according to the US Food and Drug Administration [19], if a drug product contains a drug substance that is chemically identical and is delivered to the site of action at the same rate and extent as another drug product, then it is equivalent and can be substituted (switchable) for that drug product. Methods used to define bioequivalence in orally administered drugs as stated by the FDA rules [20, 21] are pharmacokinetic studies in healthy patients. Consequently, the present study conducted in dogs in accordance with FDA and EMA rules clearly demonstrated the switchability of Atopica® soft capsules with Cyclavance® oral solutions when dosed orally at 5 mg/kg of bodyweight.

## Conclusions

This study demonstrates the bioequivalence between Cyclavance® oral solutions and Atopica® soft capsules. Since determining bioequivalence guaranties the interchangeability of the brand-name and generic drugs, it can be concluded that a dog taking one formulation will be able to change to another that will provide the same efficacy and safety. The oral liquid formulation of Cyclavance® solution enables accurate dosage of ciclosporin and is convenient and easy to use thus favoring compliance. In conclusion, Cyclavance® oral solution represents an interesting therapeutic option in the long-term management of atopic dermatitis in dogs.

## Abbreviations

AUC0-t: Area under the curve (blood concentration versus time) between time 0 and time of the last quantifiable drug blood concentration
AUC0-∞: Extrapolated AUC
%AUC0-∞: percentage of extrapolated AUC
BLQ: Below limit of quantification
Cmax: Observed maximum drug blood concentration
HPLC-MS: High performance liquid chromatography – mass spectrometry
kel: Terminal constant rate
LLOQ: Lower limit of quantification
Tmax: Time to reach the observed Cmax
t1/2: Terminal half-life
ULOQ: Upper limit of quantification
